# Conceptualizing the Role of Individual Agency in Mobility Transitions: Avenues for the Integration of Sociological and Psychological Perspectives

**DOI:** 10.3389/fpsyg.2021.623652

**Published:** 2021-04-20

**Authors:** Lisa Ruhrort, Viktoria Allert

**Affiliations:** ^1^Berlin Social Science Center (WZB), Berlin, Germany; ^2^Department of Spatial Transformation in the Digital Age, Faculty of Spatial Planning, Dortmund Technical University, Dortmund, Germany

**Keywords:** sustainable mobility, transition, agency, social norms, norm conflict, interdisciplinary

## Abstract

With the release of the latest IPCC report, the urgency to steer the transport sector toward ecological sustainability has been recognized more and more broadly. To better understand, the prerequisites for a transition to sustainable mobility, we argue that interdisciplinary mobility research needs to revisit the interaction between social structures and individual agency by focusing on social norms. While critical sociological approaches stress the structural barriers to sustainable mobility, political discourse over sustainable mobility is still largely dominated by overly individualistic approaches, which focus on individual behavior change neglecting its social embeddedness. With discursive struggles over sustainable mobility intensifying, it becomes more urgent to better understand how structural contexts condition individual travel behavior, while at the same time showing how individuals engage in processes of social change. Against this backdrop, the article seeks to deepen the cooperation between sociological and psychological research in mobility transitions research. Building on a broad body of literature, we revisit recent theoretical approaches, which conceptualize the role of individual agency in sustainability transitions. On this basis, we highlight the role of social norms in mobility transitions as a key concept bridging individual behavior and social structures. Using Strong Structuration Theory as an integrative framework, we focus on the role of individual agency in processes of re-negotiation of social norms. Our main hypothesis is that individuals can contribute to mobility transitions by influencing and re-negotiating social norms, especially in the context of windows of opportunity. We analyze how focusing on the dynamic and conflicted nature of social norms can help to illustrate leverage points for a mobility transition as well as inspire future empirical research in the field. This includes that individuals can influence social norms through changing their own travel behavior as well as through engaging in discourse on transport policies.

## Introduction

With the release of the latest IPCC report and the first indications of climate change becoming visible in central Europe, the urgency to steer the transport sector to ecological sustainability has been recognized more and more broadly (Verkehrswende, 2018). The German government has set itself the goal to reduce transport emissions by 40 percent by 2030 (BMU, 2019). As scenario studies have shown, this goal cannot be reached by switching to zero emissions vehicles alone; climate neutrality requires a modal shift from private cars to more efficient modes of transport and an overall reduction in travel demand (Zimmer et al., 2016). In this sense, a sustainability transition in the transport sector equals a disruption of current trends: for decades, the number of cars as well as overall travel demand in Germany have been growing continually (Nobis and Kuhnimhof, 2018).

To better understand the prerequisites for a large-scale modal shift to more sustainable transport modes, we argue that interdisciplinary mobility research needs to revisit the interaction between social structures and individual agency. With discursive struggles over sustainable mobility intensifying, it becomes more urgent to better understand how structural contexts influence and condition individual travel behavior, while at the same time showing how individuals engage in processes of social change. A promising way to achieve this is to deepen the cooperation between sociological and psychological research (Upham et al., 2020). Recently, critical sociological approaches have stressed the structural barriers to sustainable mobility in the context of a capitalist system of production and consumption (Dörre, 2019, 2020; Mattioli et al., 2020). Yet this perspective can obscure the role, which individuals might play in fostering a transition to sustainable mobility. By contrast, the political discourse over sustainable mobility is still dominated by overly individualistic approaches, which focus on individual behavior change, while neglecting its social embeddedness. While this perspective has been criticized extensively (Shove, 2010; Barr, 2015; Göpel, 2016), there is an ongoing tendency of mainstream political strategy to locate responsibility for a mobility transition mainly on consumer decisions. Psychological research has developed a broad array of theoretical concepts, which account for the social embeddedness of individual behavior change (section “The role of the individual in sustainability transitions”; Göpel, 2016). In this paper, we revisit some of these and look at the potential intersections with systemic accounts of socio-technical change found in sociological research. In this approach, we can build on a substantial body of literature, which has explored different avenues of cooperation between the two disciplines in the field of transition studies (Upham et al., 2015b, 2019; Bögel et al., 2019). On this ground, we propose to focus on the role of competing social norms to better understand the mutual influence of individual agency and social structures in mobility transitions. While the concept of “sustainable mobility” includes multiple dimensions (Banister, 2008), the article focusses on the goal of reducing the modal share of trips made with resource intensive modes, especially driving and air travel. The remainder of this article is structured as follows: Section “Background and problem description: Stability and change in the socio-technical system of mobility in Germany” draws on the example of Germany to briefly show the lack of progress in achieving ecologically sustainable mobility, but also some “cracks” in the established socio-technical regime of mobility. Against this background, section “The role of the individual in sustainability transitions” presents theoretical approaches, which bridge the gap between structure and agency in sustainability transitions research (STR). In section “Connecting critical sociological theory and psychological perspectives: studying the contestation and re-negotiation of social norms,” we draw on these approaches to develop our main hypothesis: a key avenue for joint sociological and psychological research in mobility transitions lies in studying competing social norms. Section “Conclusion” points out the limitations of this article and proposes topics for further research.

## Background and Problem Description: Stability and Change in the Socio-Technical System of Mobility in Germany

Reducing car-based mobility, and flying, is seen as an essential part of sustainability strategies in the transport sector (Zimmer et al., 2016; Verkehrswende, 2018). Yet, while achieving a modal shift and encouraging the use of more sustainable modes has been a long-time goal, little progress has been made so far (Schwedes, 2011). In the example of Germany, both transport demand and the number of cars on the road are growing, with roughly 75 percent of miles being traveled by car (Nobis and Kuhnimhof, 2018). Safeguarding the growth of the automobile industry, which employs around 800.000 people, is a central goal of the German federal government (Canzler and Knie, 2018). Public transport as well as cycling and walking play a major role in everyday mobility too, but are far less dominant in terms of their corresponding economic structures and political representation. Despite these strong path dependencies, recently some “cracks” in the established structures have begun to appear (Ruhrort, 2020). In many larger cities, the modal share of car trips has stagnated or has been slightly reduced, the modal share of cycling has increased, public transport demand has been stabilized, and new mobility services have emerged (Gerike et al., 2020). Also, the “cultural hegemony” (Brand and Welzer, 2019) of the car seems to have become somewhat contested: since 2016, several cities saw successful initiatives for cycling referenda (Von Schneidemesser, 2021), and the years 2018 and 2019 were marked by a growing societal awareness for climate change (Gössling et al., 2020).

From the transition research perspective, the mobility sector in Germany, while being marked by strong path dependence, has thus begun to show some signs of destabilization. Especially in the years 2018/2019, potential pathways for substantial change became visible: with large numbers of people temporarily joining climate protests or advocating for the replacement of car infrastructures with cycling infrastructure in many cities, dominant concepts of “normality” in the transport sector temporarily appeared to be losing some ground. In the language of transition theory, this situation could be characterized as a *window of opportunity* for change in the direction of sustainability. According to Geels et al. (2018), windows of opportunity can be seen as moments of intensified struggle between established structures and alternative options. In this context, the question of the interaction between social structures and individual agency for socio-technical transitions in mobility becomes particularly relevant: can individuals play a role in intensifying change dynamics? Or are the constraints posed by dominant social structures too strong to overcome? While previous research has already identified different ways in which social psychological perspectives can be integrated into mobility transitions research (Whittle et al., 2019), we will focus specifically on the role of social norms in a recursive relationship between structure and agency. As Whittle et al. (2019) point out, individual mobility related behavior often reproduces dominant social norms, but may also contribute to shifting social norms (Whitmarsh, 2012). We draw on Strong Structuration Theory to elaborate on the way in which individual agency can contribute to shifting social norms relating to travel behavior in the context of everyday life.

## The Role of the Individual in Sustainability Transitions

### Structural Barriers to Individual Behavior Change: Contributions From Critical Sociological Perspectives

As several critics have noted, mainstream political discourse tends to misconstrue the role of individuals by locating responsibility for a mobility transition mainly on the level of individual consumers’ mode choice and vehicle purchase decisions (Shove, 2010; Marsden et al., 2014; Barr, 2015; Verkehrswende, 2019). This perspective refers to economic concepts of individual choice and a selective consideration of psychological research exploring the intra-individual factors, which influence the willingness to switch from less to more sustainable options. Although psychological research and interdisciplinary approaches from transition studies have developed various approaches to study the role of individual-level action in the field of sustainable mobility (Whittle et al., 2019), the dominance of individualistic models of behavior change in mainstream political discourse still often obscures the surrounding social structures like dominant societal norms and expectations, which set limits against ecological behavior (Schwanen et al., 2011). Göpel (2016) attributes this focus on an individualistic model of change to political convenience: trying to motivate individuals to make “better choices” allows political actors to avoid confrontation of powerful interests. In addition, this strategy can help to skirt conflicts between different political goals such as economic growth and ecological sustainability (Schwedes, 2011; Marsden et al., 2014; Göpel, 2016).

On the other hand, a rich body of literature from sociology and human geography, has highlighted the role of social structures, e.g., in the form of shared practices, institutional settings, and power relations to explain the persistence of ecologically unsustainable travel behavior (Götz et al., 2016; Manderscheid, 2020; Mattioli et al., 2020). Recently, critical approaches from different social sciences have doubled down on this by stressing the structural barriers to a sustainability transition in the transport sector. For example, Dörre (2020) argues that the ecological crisis caused by growing emissions in the transport sector needs to be seen in the context of multiple crises, which are triggered by the inherent tensions of capitalist market systems. From this perspective, growing transport demand is a symptom of a system of production and consumption, which is dependent on continuous economic growth and expansion (Schwedes, 2017). Ecologically conscious behavior, e.g., buying fewer cars, would directly challenge the foundation of this model of growth, especially in Germany, where the automobile industry is focused on building luxury cars (Canzler and Knie, 2018). From the perspective of cultural sociology, Rosa (2005) sees the continuous growth of consumption (and thus the ecological “footprint”) in modern societies as the expression of a culture of *acceleration*. In his view, modern society is characterized by imperatives of growth, which, at the individual level, are experienced as social norms of constant self-optimization and self-expansion (Blättel-Mink, 2020). In this perspective, growing transport demand results from societal norms, which demand individual maximization of opportunities. Individuals feel the pressure to make the most of the opportunities presented to them: consuming as much of the world as possible (Rosa, 2016). Deviating from this norm, e.g., by seeking slower modes of living or by renouncing opportunities to travel, faces high barriers (Paech, 2019).

Similarly, Brand and Wissen (2018) describe the dominant lifestyle of Western societies as an *imperialistic lifestyle*, which “normalizes” resource intensive consumption such as car use in the form of dominant social representations of “the good life.” They also stress that the structures of the dominant growth-oriented economic paradigm express themselves in the form of a *hegemonic discourse*, conceptualized as a coherent set of social representations and norms explaining why the current patterns of production and consumption should be preferable to possible alternatives. This hegemonic discourse is often influenced by the interests of those social groups who benefit most from the status quo (Feola, 2020). Göpel (2016) follows up on this by exploring the role of dominant paradigms, which have shaped societal discourse regarding the role of individuals in modern capitalist societies. According to Göpel (2016), the dominant discursive paradigm of the role of individual actors in society is shaped by neo-classical economic theories, which conceptualize individuals mainly as market participants focused on maximizing their individual self-interest. Driven by potentially insatiable desire for consumption (e.g., in the form of cars, holiday trips, etc.), this discursive representation of the *homo oeconomicus* is conceptualized as a perfect match to a system of production and accumulation, which depends on unlimited growth. As Göpel (2016) points out, this paradigm has not only dominated academic economic thinking, but has also been instrumentalized politically to become the dominant conceptual framework of understanding society and individual agency in many political fields. “Normal” behavior has thus been equated with an orientation toward ever-increasing consumption.

The critical social scientific perspectives presented here can give insights into the barriers to sustainable travel behavior. They stress that ecologically unsustainable mobility practices are deeply embedded in the fabric of “normal” consumption patterns. Instead of building on individual behavior change, these perspectives stress that a transition to sustainable mobility needs to be achieved through political processes and struggles. Following this argumentation, it can be hard to see how individual behavior can play any part in contributing to sustainability transitions. In stressing the long-term stability of social structures these approaches also do not spell out how systemic dynamics in the form of windows of opportunity can change the conditions for individual level action. To bridge this gap, the following sections present recent theoretical approaches, which identify intersections between structuralist accounts and individual level agency and seek to apply these approaches to mobility transition research.

### The Multi-Level Perspective as a Framework for Connecting Analytic Levels

One of the most prominent frameworks to study interactions between different societal levels in sustainability transitions is the Multi-Level-Perspective (MLP) on socio-technical transitions (Geels, 2002). The MLP has increasingly been used to study sustainability transitions, also in the transport sector (Geels, 2012; Whitmarsh, 2012). At the center of this concept is the idea that socio-technical systems, such as the automobile system, are stabilized in the form of a socio-technical regime, which is marked by high (dynamic) stability and strong path dependencies, meaning that radical changes are difficult to achieve. Despite this high stability, socio-technical regimes can come under pressure from two sides: on the one hand, the broader societal environment, called landscape, constantly changes and can threaten the stability of regime structures (Geels et al., 2018). On the other hand, niche actors can try to challenge the regime by introducing innovations. It is often difficult for the latter to break through into mass markets, because the institutional structures of the regime are designed to support the dominant technological solutions (Geels, 2014). Under certain circumstances, multi-level dynamics can open up windows of opportunity, which allow niche innovations to gain momentum and threaten the dominant regime, leading to changes in regime structures or to the establishment of a new socio-technical regime.

Recently, MLP-scholars have specifically explored the possibilities of using the framework to study interrelations of structure and agency in change processes (Bögel et al., 2019). Elaborating the micro-structures inherent in the MLP, Geels (2020) points out that, while the framework has often been applied with a macro-level perspective of socio-technical change, it is not *per se* a structuralist approach. Having its roots in the Social Construction of Technology (SCOT) framework, it lends itself to studies of the role of individual agency in innovation processes. As Geels (2020) points out, SCOT-approaches tend to “follow the actors” and try to understand how strategic action of social groups, firms, or individuals help to bring about the breakthrough of specific innovations. Yet, as Bögel and Upham (2018) show, in the application of the MLP, agency has often been analyzed with regard to meso-level actors such as firms or organizations, while the role of individuals as consumers or citizens has received less attention in this research tradition (Whitmarsh, 2012; Whittle et al., 2019). Recently, Göpel (2016) has proposed to expand the three levels described by the MLP by adding a dimension of individual level action highlighting how individuals can influence transition processes in multiple ways as they adopt different roles within society. She describes this “mini” level as a realm strongly structured by macro-level cultural paradigms and dominant mindsets [e.g., in the form of dominant norms of consumption such as buying a sport utility vehicle (SUV) or taking overseas holidays], which influence individual level action. Yet, she also attributes the potential to individuals to become aware of and questions these dominant paradigms (ibd.).

### Psychological Approaches to Conceptualizing the Role of Individual Agency in Mobility Transition

Alongside integration of individual agency of Göpel (2016) into the MLP, several scholars underlined the importance of a differentiated view of individuals in transition processes (Whitmarsh, 2012). Nielsen et al. (2021) distinguish five roles in which individuals can contribute to societal change: as consumers, as investors or producers, as participants in organizations, as members of communities and as citizens. Psychological research can explain the intra-individual factors and group processes motivating agency associated with these different roles (Upham et al., 2020). Transition research can make use of these psychological theories to get a nuanced understanding of the actor perspective as Upham et al. (2020) have illustrated in their conceptual and empirical work (Bögel and Upham, 2018).

A key question in mobility research, focusing on the individual as a consumer, addresses mode choice. Environmental psychologists have explored the motives for choosing a particular mode of transport and potential barriers to changing it (Hoffmann et al., 2017; Taube et al., 2018). These studies draw on different approaches such as the Theory of Planned Behavior (Ajzen, 1991) describing mode choice mainly as an intentional decision process or conceive mode choice as a habitual behavior, to name only some of the prominent conceptualizations (Hunecke, 2015; Chng et al., 2018). The literature on mode choice will not be described here in further detail (see, e.g., Chng et al., 2018 or Javaid et al., 2020 for an overview), but it is important to note that some critique commonly used behavioral models of not sufficiently mirroring the context in which the individual action is embedded (Shove, 2010). However, in line with Bögel et al. (2019), we argue that there are social-psychological approaches explicitly addressing the influence of social and structural factors and thereby acknowledging the complexity of individual behavior. Through the concept of social norms, one can study the influence of social and structural factors, assuming that power structures, cultural characteristics, and shared mind-sets are manifested in normative beliefs. Social norms are “unspoken rules” (Barth et al., 2016), typically shared within a certain referent group. One can differentiate between *descriptive norms*, which refer to “what group members commonly do” and *injunctive norms*, which refer to what is commonly approved and disapproved of a particular group. The impact of social norms in environmental behavior is well documented for, e.g., recycling and water or energy conservation behavior (Cialdini and Goldstein, 2004; Fielding and Louis, 2020). In the context of mobility research focusing on the consumer role, there is evidence for the influence of social norms on, e.g., electric vehicle adoption (Barth et al., 2016) as well as on self-reported travel behavior (Kormos et al., 2015; Bamberg et al., 2020). Whittle et al. (2019) combine these insights from social psychology with sociological approaches into a multi-level perspective, while investigating barriers and drivers of individual adoption of mobility innovations. They highlight how factors such as perceived trust in new technologies as well as social norms, but also infrastructures jointly influence user choices. At the same time, the authors point out that user can play a role as “social actors” who “embody and augment social norms around adoption and domestication of new vehicle technologies and modes” (Whittle et al., 2019, p. 313).

As stated above, social norms as a form of social influence are embedded in our social communities (Sparkman et al., 2020). Theories like the Social Identity Theory (Tajfel and Turner, 1986) help to explain normative influence and norm salience in a particular situation highlighting the importance of “behaviorally relevant ingroups” (Fielding and Louis, 2020). Fritsche et al. (2018) illustrate the significance of social norms in predicting environmental action in their Social Identity Model of Pro-Environmental Action (SIMPEA). Together with other social identity processes like ingroup identification, collective efficacy beliefs, and group-based emotions, ingroup norms and goals influence the appraisal of and the behavioral response to an environmental problem. These norms become salient in specific situations especially through social comparison, be it the comparison to another group, a temporal comparison within the in-group’s behavior or a comparison of one group member to the average group behavior. Psychological mobility research also focuses on the individuals’ roles as citizens or members of communities, e.g., when investigating the acceptability of transport policy measures as well as civic engagement for change (Schade and Schlag, 2003; Gehlert, 2008; Schuitema et al., 2010; Besta et al., 2018). Here too, social norms and a common social identity proved to be important factors in motivating action (Becker et al., 2020). The Social Identity Model of Collective Action (van Zomeren et al., 2008), which was adapted by Rees and Bamberg (2014) to study collective environmental action, focuses on civic engagement in initiatives as an important driver to reach the necessary degree of societal change. In mobility research, social identities refer mostly to mode of transport-related identities, environmental identities, or local identities explaining mode choice as well as acceptance of transport policy measures (Murtagh et al., 2012; Götting and Becker, 2020).

As Social Identity Theory states, individuals are simultaneously part of different social groups, which might lead to conflicting norms and goals of the different referent groups of one individual. McDonald et al. (2014) investigated how individuals react when facing conflicting norms between different social groups and found that this ambiguity can highlight the need for action for individuals (signaling: “In this ambiguous situation, my contribution might actually make a difference”). Whether this motivating effect of normative conflict translates to mode choice, support for relevant traffic policy measures or civic engagement in the context of mobility transitions, still needs to be tested. Normative conflict can not only appear in competing norms between different groups, but also as a discrepancy between a dominant descriptive norm and the injunctive norm. This is particularly common for environmental issues, where the injunctive norm often is the sustainable one competing with a dominant (unsustainable) descriptive one (Sparkman et al., 2020). In a study on local mobility culture, defined as injunctive norms concerning the design of the local transport system, Bamberg et al. (2020) observe conflicting norms in a perceived consensus to support both a multimodal mobility culture as well as perceived consensus to keep privileges of a car oriented mobility culture. As these studies show, social norms are constantly competing as discrepancies between different normative beliefs can occur on multiple levels. As humans constantly seek to reduce ambiguity, the confrontation with conflicting norms opens up opportunities for an individual to choose to act in line with the marginal norm and thereby challenging the status quo. At the same time, normative conflict can also discourage behavior change, as individuals do not have to fear social sanctioning, if there is some disagreement about a certain norm (Fielding and Louis, 2020).

Evidence suggests that social influence is an important factor in both motivating different forms of agency (especially motivating collective action like, e.g., participation in a local mobility initiative) as well as hindering change (e.g., difficulties in challenging the dominant unsustainable norm of frequent car use). Focusing explicitly on how changing normative influence plays out in mobility transition processes seems crucial. Ultimately, investigating social norms allows highlighting interdependencies between individual behavior and social structures.

### Strong Structuration Theory as a Bridge Between Individual Agency and Social Structure

Social scientific research on sustainable mobility transitions also has developed a range of approaches to studying the interconnections between individual travel behavior and social structures, e.g., in the concept of “mobility cultures” (Götz et al., 2016) as well as through the lens of mobility biographies (Rau and Manton, 2016). In transition research more broadly, Upham et al. (2015a) have explored theoretical approaches bridging sociological and psychological research perspectives, including *via* Social Representations Theory as well as Social Identity Theory (Levidow and Upham, 2017). While acknowledging that interdisciplinary integration can come with tensions between underlying disciplinary paradigms, Upham et al. (2015b, 2020) have stressed the fruitfulness of such integration. To highlight that individual agency can also influence social structure in a recursive relationship, Upham et al. (2018) build on structuration theory as developed by Giddens (1986) and elaborated in the form of “Strong Structuration Theory” by Stones (2006) as a bridge between sociological and psychological approaches (see also Upham et al., 2019). Focusing on individuals in their professional roles in institutional contexts, they study the role of individual agency in niche innovation trajectories. Upham et al. (2018) study how psychological factors such as beliefs and attitudes toward niche innovation are shaped by experiences in specific policy environments and how these “internal structures” shape the individuals’ expectations and, ultimately, their actions in regard to the innovation. Following Stones (2006), they conceptualize a dualistic relationship: individual action is conditioned by external social structures such as norms, value systems, and shared social practices. These are seen as the (intended or unintended) result of previous actions. Stones (2006) stresses that external social structures match internal structures in the form of “conjunctural knowledge” and general dispositional structures (“habitus”), which individuals draw on to participate in social practices. By drawing on these structures to guide and enable their actions, individuals are constantly engaged in reproducing these structures, ensuring their stability over space and time.

Importantly, social structures, just like material infrastructures, fulfill a double function of both constraining but also enabling specific paths of action. From a transition perspective, it is important to note that both Stones (2006) and Giddens (1986) stress the potential role of individual actors in bringing about social change. While social structures are powerful in shaping individual actions, humans always have the option of switching from the *practical consciousness* of everyday life, in which underlying structures are not questioned, to a state of “reflexivity” (Giddens, 1986). In this state, individuals can act in different ways and also challenge social norms or practices (see Archer, 1995). In addition, Stones (2006) stresses that the relation between internal and external structures but also between different elements of internal structures such as normative beliefs, can be marked by substantial tensions. Individuals are constantly challenged to manage a “plurality of concerns” (Stones, 2006, p. 103), which necessitate flexible prioritization. In each situation “choice [e.g., between different norm prioritizations] is possible, even mandatory, because more than one course of action has systemic legitimacy” (Stones, 2006, p. 105). Individuals are thus not conceived as “cultural dopes” who reproduce normative expectations and rules, but as skillful actors who constantly negotiate between conflicting orientations. From the perspective of mobility transitions this concept highlights the constraints to more sustainable travel behavior in the form of dominant descriptive norms, but also points out how already existing tensions between different internal normative orientations might harbor the potential for change. In this way, Strong Structuration Theory highlights that individual level action can contribute to changes in social structures by influencing social norms.

As this section has shown, there is a substantial body of literature, which explores intersections between sociological and psychological perspectives in transition research. In line with that research, we argue that social structures in the form of collectively shared concepts of “normality” strongly condition individual mobility-related behavior and pose substantial barriers against behavior change. At the same time, we argue that individuals have the capacity to challenge social norms and contribute to social change. In this context, we want to highlight an aspect of social norms, which may be of particular importance in the context of beginning change dynamics, namely struggles between conflicting social norms.

## Connecting Critical Sociological Theory and Psychological Perspectives: Studying the Contestation and Re-Negotiation of Social Norms

### Re-Negotiations of Social Norms of Travel Behavior in the Context of Windows of Opportunity

The analysis above has shown that one intersection between sociological and psychological approaches lies in the concept of social norms, which guide and influence both individual (travel) behavior and civic engagement in transition processes. Building on the differentiation between descriptive and injunctive norms (Kallgren et al., 2000; Barth et al., 2016), we suggest that joint research in the transport sector should focus more explicitly on social norms as conflicting and contested. In the course of transition dynamics, tension can increase between injunctive and descriptive norms as well as between descriptive norms in different social groups or between different spatial settings such as urban and rural settings. For example, recent years have seen shifts toward increased use of alternatives to the car in cities (e.g., descriptive norms relating to cycling and PT-use), while daily travel behavior in suburban communities have remained strongly car-dependent (descriptive norm of monomodal car-use; Nobis, 2019). On the level of political discourse this is expressed in intensifying political debates over the role of the car in local transport policy in many cities (Becker et al., 2020) and increasing tensions with the interests of car-users in the suburbs (Henderson and Gulsrud, 2019).

Such tensions are not unusual. Individuals in modern western societies are constantly confronted with competing norms resulting from different frames or groups of reference (Beck and Beck-Gernsheim, 1994; Stones, 2006; McDonald et al., 2014). This may especially be true for those norms, which are central to sustainability transitions. As the sociological approaches above have shown, ecological behavior is currently not the (dominant) social norm in our society. Brand and Wissen (2018) point to an “imperialistic lifestyle,” which normalizes the consumption of energy intensive products and services such as cars or flying. Gössling (2019) shows how flying is traditionally highly “charged” with symbolic meaning as an expression of high social status. Against this backdrop, ecological behavior, if it goes beyond “low cost” behavior such as recycling, represents a deviation from dominant descriptive norms, while constituting support for a set of competing niche norms. Not buying an SUV can be deviant behavior – if all neighbors own one; not taking a flight to go on holiday can be deviant behavior – if most friends and family members regularly take overseas holidays (Gössling et al., 2020).

Especially when problems such as climate change come to the forefront in public and media discourse, individuals are increasingly confronted with tensions between contradictory norms. This has recently been the case in the transport sector in Germany. The rise of debates around climate change and the need to adapt more sustainable lifestyles (injunctive norms; Hessenschau, 2019), combined with growing levels of cycling and public transport use in some cities (descriptive norms) have strengthened alternative descriptive and injunctive transport-related norms in societal discourse (Bamberg et al., 2020; Dörre et al., 2020). From a sociological perspective, we can conceptualize these systemic dynamics as struggles between dominant norms and alternative niche norms in the context of a socio-technical transition process. As was visible in Germany in 2018/2019 key elements of a “hegemonic discourse” in mobility such as the role of the car in socially dominant concepts of “the good life” were beginning to be debated. Policy measures such as car-free city centers or congestion charges, which used to appear unacceptable for a majority, were suddenly being debated in media discourse and private settings (Andor et al., 2020). In this situation, contradictions between competing norms, such as the descriptive as well as injunctive norms of environmentally conscious lifestyles and unsustainable travel behavior (e.g., taking long-distance flights) became more salient.

From a systemic perspective, this situation can be seen as an example of a window of opportunity for change. Systemic models of socio-technical transitions suggest that the odds to achieve change are dependent on the historical and systemic context, in the form of windows of opportunity, but also positive feedback loops and tipping points (Urry, 2004; Watson, 2012; Ruhrort, 2020). With reference to the extended version of the MLP as proposed by Göpel (2016), we suggest that for individual level agency to effectively support sustainability transition processes may strongly depend on system dynamics. In a window of opportunity, norms and routines of prioritization become destabilized and contested. This effect is often mirrored in political discourse (e.g., parties scrambling to readjust their agenda to what might be changes in public opinion); but also in personal social contexts, e.g., in the interaction with work colleagues, friends, or family members. Some ideas or concepts of normality become open for re-negotiation (Whitmarsh, 2012; Nash et al., 2020).

In a window of opportunity, we argue that individuals in their role as consumers and citizens can contribute to change by engaging in the re-negotiation of social norms, both in their everyday practices as well as in the political realm. Individuals can influence social norms by engaging in a specific behavior, especially when this behavior is visible in social context. Choosing to cycle to work once a week can influence the normative beliefs held by work colleagues regarding cycling and its acceptability as a mode choice for a commute. Choosing to bring the children to school by bike instead of by car, even though this is not the dominant norm, can initiate changes about the perceived normality of this mobility practice. When norm-conflict becomes salient, individuals can contribute to the already ongoing change dynamics by becoming vocal and active, e.g., by performing symbolic acts of consumption, which are shared in private interaction or on social media in the context of organized platforms (e.g., by stating: “I decided I will not fly to go on holiday for the next 3 years”; Gössling et al., 2020).

### Conceptualizing the Recursive Relationship Between Social Norms and Agency as a Process of Structuration

Sociologically speaking, in a window of opportunity there is a heightened chance that such actions will have a cumulated effect on changing social norms or opening up pathways for the implementation of decisive policy measures. Gössling et al. (2020) find evidence that social movements, especially Fridays for Future, successfully influenced social norms regarding flying, re-defining air travel as a morally problematic social practice. While their study focuses on the role of social movements in shifting social norms, other recent examples also show how individuals as consumers can participate in reinforcing and stabilizing such ongoing shifts. For example, in 2019 thousands of individual scientists joined an international initiative by signing a public pledge to renounce air travel on academic trips below 1,000 km (Nietfeld, 2019). This type of symbolic action can help to de-legitimize a dominant social practice and re-negotiate the underlying social norms through their own behavior change (Gössling et al., 2020). It can be seen as an example of how individuals can choose to forego the reproduction of descriptive norms (flying) and thus can contribute to changing these norms themselves. Beyond air travel, similar tapes of symbolic action could be possible in the realm of every-day mobility: e.g., when car-users decide to cycle to work at least once a week even though this practice is deemed unusual among colleagues or neighbors; or when a resident in suburban community decides to express dissent about car-related norms (e.g., by stating “My child struggles navigating his way to school, when there are so many parents parking their cars in front of the school entrance”) in a conversation among neighbors.

**Figure 1:**
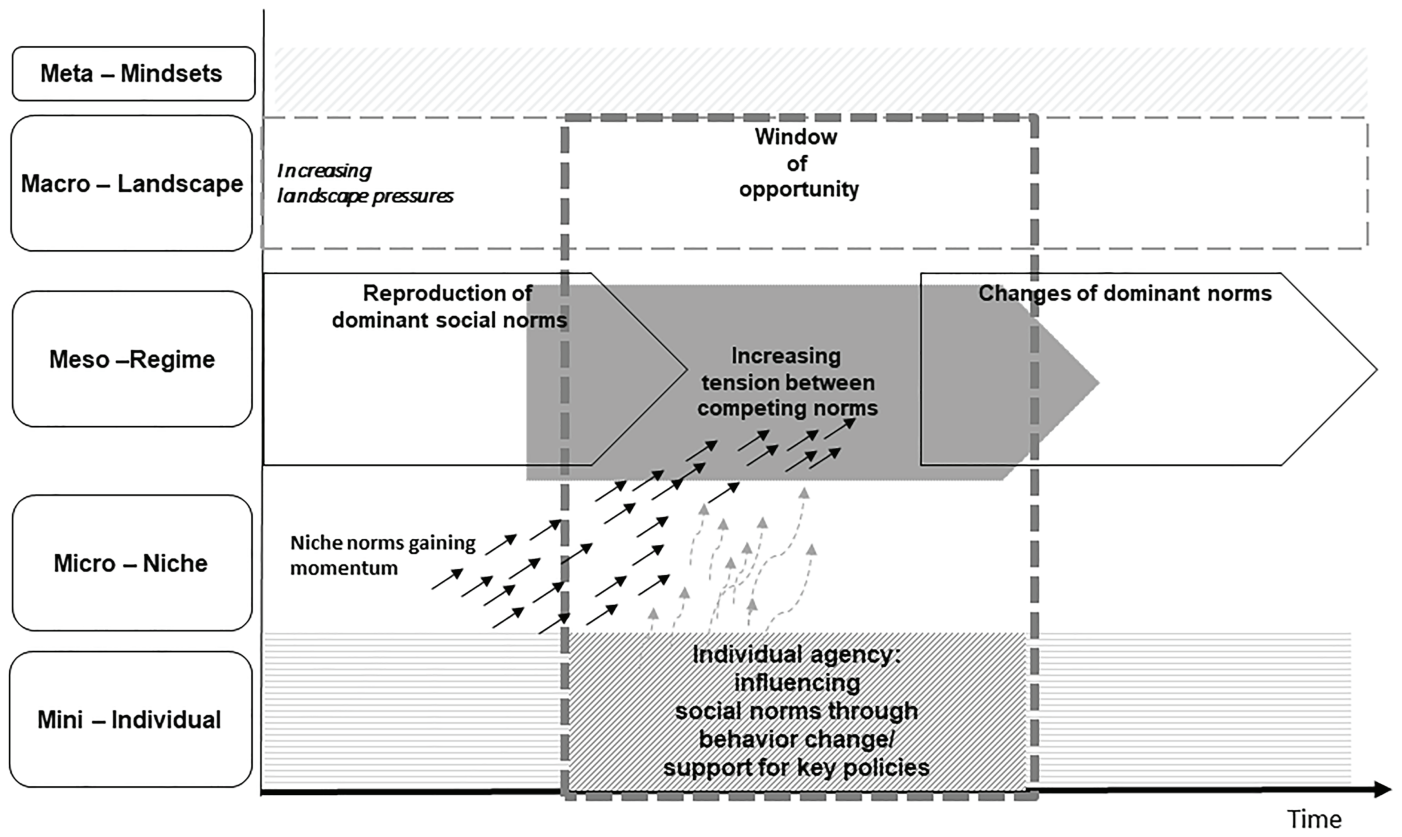
Individual agency in the context of multi-level system dynamics (based on Göpel, 2016 and Geels et al., 2018).

On a theoretical level, this opportunity for re-negotiation of norms can be understood as an element of a cycle of structuration. Following Upham et al. (2018, 2020), Strong Structuration Theory can explain the reproduction of social structures through individual action, while also pointing out the often contradictory nature of social norms and highlighting opportunities for change (Stones, 2006). Concerning beginning change dynamics in the mobility sector, we suggest to focus on the temporal dynamics of contradictory norms: individuals are regularly confronted with multiple norms and need to take decisions (reflexively or unconsciously) to prioritize some norms and expectations over others (Stones ibd.). The more ambiguous the normative context becomes, the more individuals may become aware of multiple courses of “normal” or “legitimate” action. Following the cycle of structuration conceptualized by Strong Structuration Theory also highlights the (intended or unintended) outcomes of the courses of action chosen by agents. Individual deviance from dominant norms can interrupt the reproduction of “normal” practices and can thereby initiate changes in social norms (see Figure 1). In the language of Strong Structuration Theory, individuals can decide to act in line with alternative norms.

The examples mentioned above illustrate how individual behavior change can influence social norms. The main contribution individuals can make thus might not be in its direct effects (e.g., CO_2_-emissions reduced) but in its indirect effect on changing descriptive norms (Whitmarsh, 2012). As we will illustrate in section “Studying contested norms and processes of re-negotiation: Open questions for empirical research,” to better understand the concrete processes of re-negotiation in the mobility sector psychological and sociological research could be integrated in the form of local case studies. Sociology can study different practices and varying contexts (i.e., social media, private conversations, symbolic acts of consumption etc.) of re-negotiation (Gössling et al., 2020). Psychology can study the determinants for individuals’ willingness to deviate from unsustainable norms as well as the individual perception of norms and their situational salience.

### Studying Individual Agency in the Collective Re-Negotiation of Social Norms

Even though individual behavior change in this way can make an important contribution by influencing social norms, it is important to note that this type of change alone will probably not suffice to bring about the level of systemic change needed. As stated earlier, for substantial changes in the mobility system, far reaching regulatory and institutional changes are also required. As Ruhrort (2020) argues, large-scale change of travel patterns can only become possible if infrastructures are re-designed to suit the needs of active travel modes, the regulatory framework is changed to roll back the privileges afforded to private cars and pricing modalities reflect external costs of different modes. Importantly, this means that sustainability transitions are not necessarily a win-win-process, but will raise the key political questions of “*who gets what, when, and how*” (Lasswell, 1936). “Pull measures,” which make transport alternatives more attractive will have to be accompanied by “push measures,” which are aimed at reducing the attractiveness of cars and other resource intensive travel modes (Ruhrort, 2019). When transport policy measures go beyond “win-win”-approaches formerly dominant injunctive norms guiding transport policy become acutely challenged (Bamberg et al., 2020).

On this level, individuals can support and initiate these change processes in their role as citizens (Whitmarsh, 2012; Nielsen et al., 2021). Policy discourse over “push measures” can be seen as a collective form of re-negotiation of what is to be considered normal in the realm of mobility in public space. In this context, individual citizens are confronted with competing sets of norms, either gradually or suddenly. As mentioned above, Bamberg et al. (2020) found substantial ambiguity in how study participants perceived the injunctive norms regarding prioritization of car mobility vs. multimodal mobility in local transport policy. This can be seen as an indication of beginning change dynamics, which could open windows of opportunity for substantial changes. Individuals have a chance to “tip the balance” toward change by actively or discursively supporting policy measures, which challenge the status quo (Ruhrort, 2019). With regard to air travel, Gössling et al. (2020) make this connection by studying not only individuals’ willingness to refrain from flying, but also their willingness to accept, or demand, policy measures, which help to reduce air travel on a larger scale. Becker et al. (2020) have highlighted the role of norms in political negotiation over transport policy “push measures” regarding the distribution of public space. They study a local NGO successfully building public support for a referendum for cycling infrastructure. The authors describe how the initiative countered the normative status quo by changing “normative associations”: by representing cycling as normal and as equally important to car travel, the initiative did not address a narrow social identity of “committed cyclists,” but instead appealed to a more inclusive social identity. According to the authors, this strategy helped to elicit support from a broader public. As with other processes of re-negotiation of social norms, the effectiveness of changes will be strongly context-dependent. Nevertheless, collective re-negotiations like discussions about the use of public space and the elaboration of new traffic policies represent an important way how individuals can make use of their role as citizens to impact the mobility transition.

### Studying Contested Norms and Processes of Re-Negotiation: Open Questions for Empirical Research

An open question regards the empirical study of the role of contested norms in enabling individual engagement in change processes. A fruitful arena for interdisciplinary research could be found in local case studies of mobility discourses and policies. As suggested by Upham et al. (2020), a sequence of disciplinary studies could trace the interactions between system dynamics and individual level action in a local context. To study how dynamics of re-negotiations of social norms play out in a local context, we suggest focusing on spaces where conflicting social norms can be expected to “clash.” Building on previous work (Bamberg et al., 2020), we propose to shift the focus to conflicting norms in a specific type of spatial setting, namely local communities at the intersection between urban and suburban spaces. Especially urban centers in Germany have seen shifts in modal shares as well as mobility related discourses, which have been identified as the emergence of a distinctive urban “mobility culture” (Ruhrort, 2019; Bamberg et al., 2020). In this context, it can be assumed that *suburban* communities, which surround the city increasingly become the locus of competing normative orientations regarding travel behavior and policy. While, we expect that in these communities, descriptive norms regarding car driving will be stronger than in the city, these communities will also be exposed to competing norms originating in the regional urban center regarding the use of other transport modes and transport policy programs. With many people commuting, individuals are exposed to different social groups potentially sharing different sets of mobility-related norms.

In local case studies, sociological analysis of system dynamics can re-construct the locally specific discourses relating to dominant and niche mobility practices and transport policy measures. Qualitative interviews could identify specific local issues in which competing mobility related concepts of “normal” practice may be “clashing”: examples could be the local “school run” and whether or not it is deemed normal to bring children to school in cars or on a bike. In this context, local examples of re-negotiations of mobility related norms could be reconstructed (e.g., if neighbors are debating over SUVs and their contribution to climate change or over the possibility to cycle to work). Psychological approaches could study how competing descriptive norms are perceived by individuals in this community and how they influence individual willingness to support (or reject) niche norms through behavior change. Following McDonald et al. (2014), a case study could measure tensions between conflicting norms as perceived by individuals. An example would be to study to which extent individuals in a suburban community perceive the dominant descriptive norm of car ownership and driving (or, more specifically, owning and driving resource intensive cars such as SUVs) as increasingly contested: do they perceive that competing descriptive norms (such as using less resource-intensive forms of mobility such as cycling) are gaining in relevance? How does the affiliation to different social groups (e.g., neighbors in the suburban community vs. work colleagues living in the city) and the potentially conflicting norms between them influence individual mobility-related decisions, e.g., the readiness to take the children to school by bike even if this is not the locally dominant norm? To encompass the political dimension of mobility transitions, the analysis should also study the support for relevant (local) transport policy measures: how are discourses over conflicting injunctive norms, e.g., regarding the redesign of street spaces, perceived by individuals in a given local or social context? How do these perceptions influence the willingness to support or accept policy measures, which aim at reducing currently dominant unsustainable travel patterns? In combining both disciplinary approaches, local case studies could show how individual motivation to participate in re-negotiation of mobility-related (local) norms through mode choice changes or political engagement may be influenced by societal discourses and practices, which de-stabilize dominant norms. Even if such multi-disciplinary research design may entail tensions between underlying disciplinary paradigms (Upham et al., 2015b), we suggest it can be fruitful to better understand interactions between different societal levels in mobility transitions.

## Conclusion

In this article, we presented intersections between sociological and psychological research, which could help to differentiate the role of individual agency in mobility transitions. The role of social norms is proposed as an integrative concept to study the interplay between structure and agency in mobility transitions. The socio-psychological approaches highlighted here have the potential to shed light on barriers to sustainable travel behavior but also on the ways in which individuals can contribute to social change in the direction of sustainability. We also highlighted that the efficacy of such individual engagement to trigger large-scale change may depend on dynamics on the system level: individual agency can play a key role especially when a window of opportunity opens up and social norms become increasingly contentious. In these situations, “social norms can spark collective action and move the needle on policy” (Hackel and Sparkman, 2018). Ultimately, socio-technical change can be stabilized if political actors and social movements can seize the opportunity to institutionalize alternative social norms by making lasting changes in mobility infrastructures and regulations.

We propose that future research should study the role of social norms in overarching models of socio-technical change more systematically. Social norms have been an element of MLP-models from the start (Geels et al., 2018), but their role has not always been at the forefront of MLP-analyses. As was shown in section “Structural barriers to individual behavior change: Contributions from critical sociological perspectives,” we propose to conceptualize social norms as conflicting and contested. In the language of the MLP, this translates into tensions between dominant sets of norms on the regime level and alternatives sets of norms, especially ecological norms, on the niche level. On the landscape level, we can identify sets of norms of a more general character, which change slowly and are not necessarily directly linked to the field of mobility (Göpel, 2016). Reformulating our analysis in the language of the MLP, we can now see that individuals, with their own behavior, have the opportunity to engage in struggles between competing social norms on the regime and niche level. Future research should explore if and how individuals can also challenge the overarching discursive paradigms, which form the normative “landscape” level of socio-technical transitions.

Beyond the academic interest, we see implications of our proposed perspective in supporting different social actors in initiating sustainability transitions. Individuals could learn to see themselves as “carriers” of social norms and practices, which they actively reproduce, but can also challenge. This understanding can encourage individuals (and potentially increase self-efficacy beliefs) to actively engage in challenging and re-negotiating social norms in their own social context. The perspective developed here may encourage individuals to look out for signs of accelerating social dynamics (e.g., in media discourse), which could become windows of opportunity for systemic change. Motivation to participate in changing social norms may be higher when individuals see themselves as effectively “pushing” a change process, which is already ongoing (Sparkman et al., 2020). At the moment, individuals in Western societies will often not be aware of these notions, a fact which can be seen as an effect of the dominance of individualistic paradigms described by Göpel (2016). Challenging these paradigms could have significant potential for triggering individual motivations to contribute to change. Ideally, socio-psychological models describing the role of the individual in sustainability transitions will become a staple in political and media discourses on climate change and mitigation strategies. There are encouraging examples of how interdisciplinary research can illustrate the role of the individual in sustainability transitions in a comprehensible way, acknowledging the interplay between individual agency and societal structures (Capstick et al., 2020). Following up on this, socio-psychological approaches could help to challenge the dominance of overly individualistic paradigms, which are in themselves a substantial barrier to social-ecological transition dynamics in the transport sector.

The article focused on the role of social norms as a concept integrating sociological and psychological approaches in mobility transitions research. One limitation of this article is that we do not spell out the empirical applications in detail, leaving this work as a task for future research. Also, our proposed research agenda strongly focuses on potential ways in which individuals can make a difference for societal and political change. Further research needs to address how these alternative sustainable “normalities” need to be supported and stabilized by changes to the institutional setting. Focusing on social norms presents an opportunity to overcome the structure-agency dualism by highlighting how individual behavior and social structure are deeply intertwined.

## Author Contributions

Both authors have contributed equally to the conceptualization and writing of the article.

### Conflict of Interest

The authors declare that the research was conducted in the absence of any commercial or financial relationships that could be construed as a potential conflict of interest.
